# Successful Endoscopic Retrograde Cholangiopancreatography (ERCP) in a Patient With Situs Inversus Totalis: A Case Report

**DOI:** 10.7759/cureus.105162

**Published:** 2026-03-13

**Authors:** Abdullah Alotiabi, Mansour Alghamdi, Abrar Oraijah, Nasmah Jaafari, Ghadeer H. Alharbi

**Affiliations:** 1 Gastroenterology Department, Security Forces Hospital, Makkah, SAU; 2 Nursing Department, Security Forces Hospital, Makkah, SAU

**Keywords:** biliary, biliary cannulation, biliary obstruction, endoscopy, ercp

## Abstract

Situs inversus totalis (SIT) is a rare congenital condition where thoracic and abdominal organs are arranged as a mirror image of normal anatomy. Albeit often asymptomatic, it can complicate procedures such as endoscopic retrograde cholangiopancreatography (ERCP) due to reversed internal orientation. We present the case of a 52-year-old man with SIT who came to the hospital with upper abdominal pain, jaundice, and pruritus, and imaging revealed a stone in the distal common bile duct. ERCP was performed in the standard left lateral position, but the scope advancement was challenging, whereby a 360° rotation was required to align with the ampulla, which appeared in mirror position. After successfully completing cannulation and wide sphincterotomy, a single stone was removed using balloon extraction. The patient recovered without complications. This case highlights how careful technique and scope manipulation can overcome anatomical challenges in SIT without needing major changes in standard ERCP protocol.

## Introduction

Situs inversus totalis (SIT) is a rare birth condition where all chest and abdominal organs are characterized by a complete mirror-image arrangement of the thoracic and abdominal organs. The prevalence is estimated to range between one in 5,000 and one in 20,000 individuals. Usually, SIT does not cause symptoms, but it can make surgery or endoscopy more difficult, especially when the organ is not the same on both sides [1].

One of the most technically challenging endoscopic procedures in patients with situs inversus is endoscopic retrograde cholangiopancreatography (ERCP). Since its introduction over four decades ago, ERCP has evolved from a diagnostic tool to a predominantly therapeutic modality for biliary and pancreatic ductal pathologies [2]. Fewer than 50 cases discussing ERCP in SIT patients are currently indexed in PubMed, underscoring the rarity of this scenario. Even though it is not easy, some reports show successful treatments, such as sphincterotomy, removing stones, widening bile ducts, and putting in stents. In most cases, the patient was lying face down, and the scope had to be turned 180° in the second part of the duodenum to see the ampulla clearly [3].

Moreover, there is no consensus on the optimal endoscopist positioning or technique in such cases. Additionally, endoscopic treatments can still work even with changed anatomy. For example, in a 65-year-old man, a normal ERCP showed two large calculi inside the bile duct, but lithotripsy did not work. Instead, clinicians employed cholangioscopy and laser lithotripsy, and it was successful [4]. In this context, we present a case of successful ERCP in a patient with SIT, emphasizing the technical challenges encountered and the strategic adaptations that enabled a safe and effective outcome.

## Case presentation

A 52-year-old man with known SIT and no heart problems came to the hospital with a few days of progressive epigastric pain. He also had nausea, vomiting, jaundice, and pruritus. On examination, he was fully awake, looked jaundiced, and had stable vital signs. There was tenderness on palpation in the epigastrium and right upper quadrant, pressing on the upper middle and right upper side of the abdomen. Laboratory findings are summarized in Table 1.

**Table 1:** Laboratory investigations

Parameter	Result	Reference Range
AST (Aspartate Aminotransferase)	186 U/L	5-34
ALT (Alanine Aminotransferase)	394 U/L	0-55
Total Bilirubin	90 µmol/L	3.4-20.5
GGT (Gamma-Glutamyl Transferase)	1000 U/L	9-36
Amylase	65 U/L	30-110 U/L
Lipase	32 U/L	13-60 U/L

**Figure 1:**
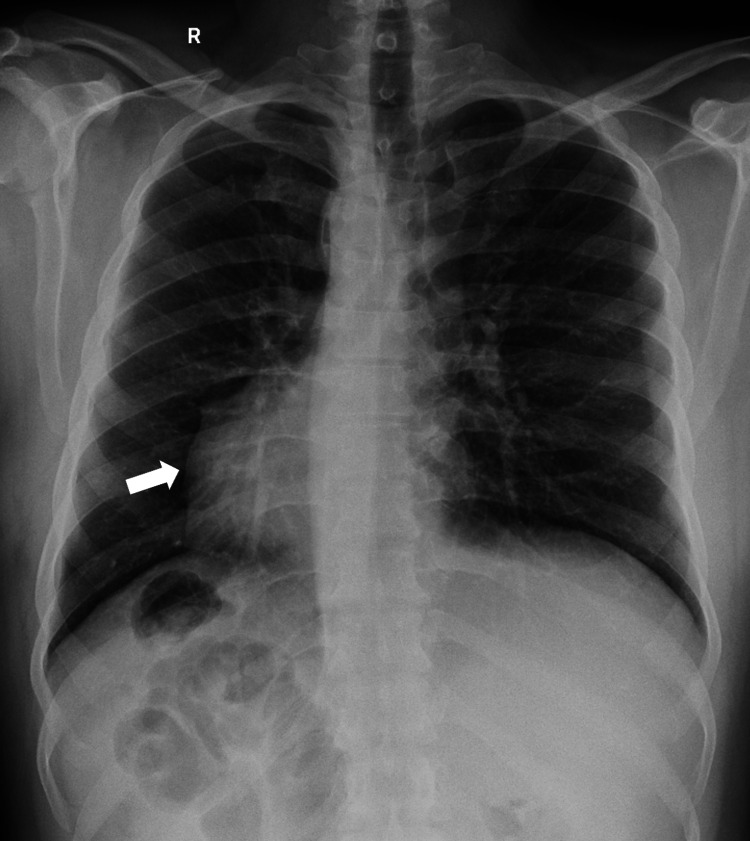
Chest X-ray showing cardiac shadow at the right

**Figure 2:**
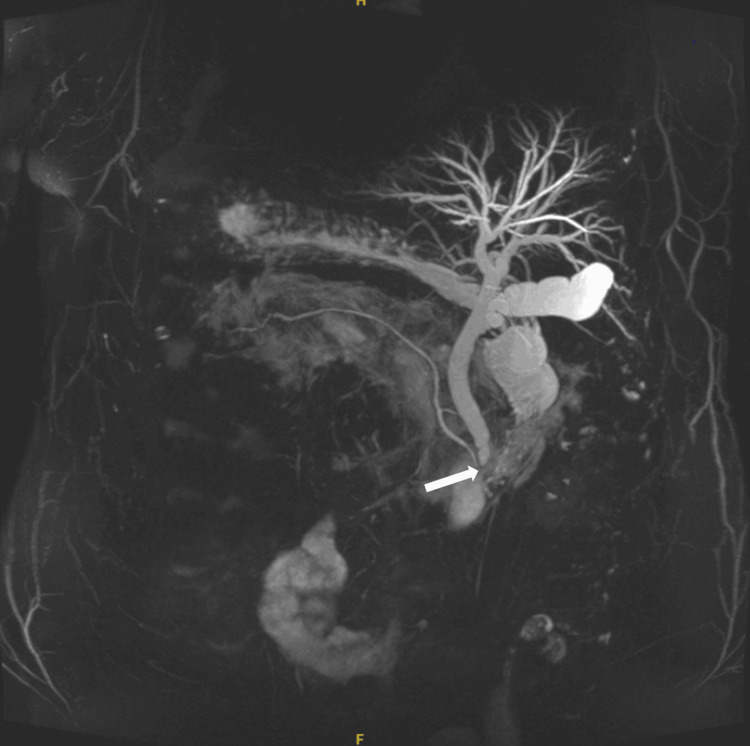
MRCP with a stone in the distal CBD CBD: common bile duct; MRCP: magnetic resonance cholangiopancreatography

A plain chest X-ray (Figure 1) showed dextrocardia. Abdominal ultrasound found gallstones, but the common bile duct (CBD) size was normal. MRCP showed mild dilation of the CBD and intrahepatic bile ducts, with a stone in the distal CBD (Figure 2).

ERCP was done while the patient lay on the left side. The scope entered the mouth, and esophageal and gastric intubation were uncomplicated, but it was difficult to reach the second part of the duodenum. We rotated the scope 360 degrees to get a good position. The ampulla looked swollen and pointed downward like a mirror image, and there was no bile flow.

After a few tries, we succeeded in cannulating the CBD without touching the pancreatic duct. Cholangiogram showed that the CBD was 9 mm wide with a small stone at the end and mild dilation of intrahepatic ducts.

**Figure 3:**
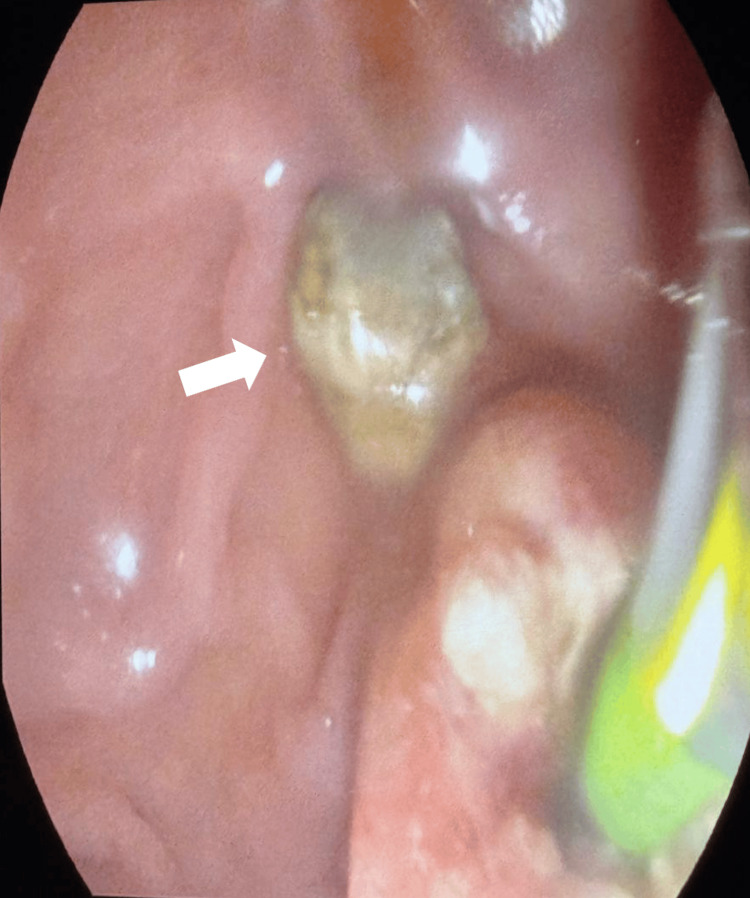
An endoscopic picture showing a hard stone (about 6-7 mm)

We made a wide sphincterotomy with no complication and then used a 13 mm balloon to achieve ductal clearance via balloon stone extraction. One hard stone (about 6-7 mm) was removed, and bile flowed well (Figure 3). The final cholangiogram showed no more stones in the CBD or liver ducts.

After ERCP, the patient had no problems, such as pancreatitis, bleeding, or perforation. His symptoms and liver test improved quickly. He was discharged and referred for laparoscopic gallbladder removal two weeks later.

## Discussion

SIT is a rare congenital condition where the chest and abdominal organs are reversed like a mirror image [3]. Although known for a long time, many doctors do not have much experience with it because it is uncommon, often seen in males. The exact frequency of SIT is hard to confirm because there are only a few direct reports and many repeated citations. Heterotaxy, which is a partial or mixed form, occurs in around 1:10,000 births [5]. The reported incidence for SIT ranges from one in 6,500 to one in 25,000 [3-5]. Because it is rare, many healthcare workers do not see it often in practice.

This reversed anatomy happens due to aberrations in embryonic development during the establishment of left-right asymmetry [5,6]. Imaging is very important to understand the exact organ position, which helps in planning procedures. ERCP is one of the most difficult endoscopic procedures in SIT patients. Because the organs are reversed, the endoscopist needs to change the way they usually do the procedure. Scope manipulation, orientation of the ampulla, and selective cannulation become more challenging due to the mirror-image anatomy. There are only a few reports about ERCP in SIT patients, so there is currently no consensus regarding the optimal technical approach [7]. In complex surgeries such as liver transplant, special techniques and flexible planning are needed to overcome the anatomical differences.

In our case, we did the ERCP with the patient lying on the left side, which is the usual position. It was not easy to reach the second part of the duodenum, but after rotating the scope 360 degrees, we could see the ampulla in a mirror-image position. Specifically, a 360-degree clockwise rotation of the endoscope was performed in the second portion of the duodenum to align with the reversed orientation of the ampulla. After some attempts, we were able to cannulate the CBD successfully without touching the pancreatic duct. The stone was removed safely after a wide sphincterotomy and balloon sweeping. There were no complications, and the patient got better quickly.

We intentionally maintained the standard left lateral position rather than changing to prone positioning. Maintaining this position preserved familiar endoscopist ergonomics and standard fluoroscopic orientation. This may reduce procedural complexity and avoid the need for repositioning the patient under sedation. In our experience, careful clockwise rotation of the scope was sufficient to compensate for the reversed anatomy without altering patient positioning.

Similar cases have been reported with varying degrees of success. Alrufayi et al. described a 50-year-old woman with SIT who had pain in the epigastric and left upper abdomen [8]. Imaging showed abnormal liver function and multiple stones in the common bile duct. She successfully underwent ERCP, with the patient lying in a supine position and the endoscopist standing on the left side. In that case, the position made it easier to reach the pylorus and duodenum and to find the papilla. The report highlighted that adjusting the patient and endoscopist positions is important in SIT to manage the mirror anatomy. This supports our case, where ERCP was also completed successfully, even though we used the standard left lateral position with careful technique and scope rotation.

Another case involved a 51-year-old man with known SIT who presented for evaluation of intermittent rectal bleeding [9]. Interestingly, the endoscopist was unaware of the SIT diagnosis during the procedure. Both upper EGD and colonoscopy were done using standard techniques without needing special maneuvers or patient position changes. The SIT was only discovered later during a liver ultrasound. This case shows that not all SIT patients need a modified endoscopic technique. However, other studies mention that SIT may cause longer cecal intubation times and recommend changes such as rotating the scope 180° in the stomach and duodenum or placing the patient in the right lateral position. In our case, although we were aware of the diagnosis before ERCP, we were still able to complete the procedure successfully using the standard left lateral position, with careful adjustment of scope handling and orientation.

Lee et al. reported a case of a 74-year-old woman with SIT who presented with epigastric pain and jaundice. Imaging showed multiple stones in both intrahepatic and extrahepatic bile ducts [10]. ERCP was performed twice using two different methods. In the first attempt, the patient was placed in the prone position with the endoscopist on the right side. The endoscope was rotated 180° counterclockwise in the stomach and again in the duodenum to reach the ampulla. In the second approach, the endoscope followed the lesser curvature while turning clockwise, which allowed smoother access.

This case demonstrates that more than one endoscopic approach may be effective for SIT patients, depending on the operator's preference and patient anatomy. Their experience highlights how technique adjustments, such as scope rotation or access angle, can improve success. Compared to their modified approach, we achieved successful ampullary access and stone extraction in the left lateral position using careful 360° scope rotation, without needing to change the endoscopist position or redo the procedure. Our case suggests that controlled clockwise rotation in the second portion of the duodenum may allow successful ERCP without routine conversion to prone positioning, provided that the operator maintains spatial awareness and careful scope control.

From a technical standpoint, several practical points may help when performing ERCP in SIT patients. First, a careful review of pre-procedural imaging is essential to understand the mirror anatomy. Second, maintaining a stable scope position in the stomach before advancing into the duodenum helps prevent loss of orientation.

## Conclusions

SIT is a rare condition that can make procedures such as ERCP more difficult due to reversed organ positions. Although SIT is inherently benign, its mirror-image anatomy presents technical challenges during ERCP. This case demonstrates that successful ERCP in SIT patients is achievable in the standard left lateral position through deliberate scope manipulation, specifically 360-degree clockwise rotation in the second portion of the duodenum, thereby potentially avoiding the need for prone positioning while maintaining procedural safety and efficacy.

Different case reports suggest that many techniques can work, such as changing the patient's position or rotating the scope in different ways. Some endoscopists prefer the supine or prone position or even standing on the other side of the patient. However, as our case and others show, ERCP in SIT patients does not always require major changes to the standard approach. This case demonstrates that successful ERCP in SIT patients is achievable in the standard left lateral position through deliberate clockwise scope rotation, potentially avoiding the need for prone positioning while maintaining procedural safety and effectiveness. It depends on the situation and the experience of the medical team. More real-world reports like ours are useful to guide doctors in choosing the best strategy for each patient with this rare condition.

## References

[1] Choi DH, Park JW, Kim BN (2008). Colonoscopy in situs inversus totalis patients. Am J Gastroenterol.

[2] Søreide JA, Karlsen LN, Sandblom G, Enochsson L (2019). Endoscopic retrograde cholangiopancreatography (ERCP): lessons learned from population-based national registries: a systematic review. Surg Endosc.

[3] Eitler K, Bibok A, Telkes G (2022). Situs inversus totalis: a clinical review. Int J Gen Med.

[4] Machado NO, Chopra P (2006). Laparoscopic cholecystectomy in a patient with situs inversus totalis: feasibility and technical difficulties. JSLS.

[5] Grimes DT, Burdine RD (2017). Left-right patterning: breaking symmetry to asymmetric morphogenesis. Trends Genet.

[6] Levin M (2005). Left-right asymmetry in embryonic development: a comprehensive review. Mech Dev.

[7] Mangan SH, Ng J, Ng J (2024). Navigating endoscopic challenges in situs inversus totalis: strategies for optimal procedure completion and patient safety. Int Surg J.

[8] Alrufayi B, Almutairi S, Zagnoon A (2025). Successful endoscopic retrograde cholangiopancreatography for management of choledocholithiasis in a patient with situs inversus totalis: a case report and literature review. Gastro Hep Adv.

[9] Yun LS, Clark A, Anderson K (2019). Endoscopy in patient with situs inversus totalis. Am J Gastroenterol.

[10] Lee JM, Lee JM, Hyun JJ (2017). Successful access to the ampulla for endoscopic retrograde cholangiopancreatography in patients with situs inversus totalis: a case report. BMC Surg.

